# Advances in Model Systems for Human Cytomegalovirus Latency and Reactivation

**DOI:** 10.1128/mbio.01724-21

**Published:** 2022-01-11

**Authors:** Lindsey B. Crawford, Nicole L. Diggins, Patrizia Caposio, Meaghan H. Hancock

**Affiliations:** a Vaccine and Gene Therapy Institute, Oregon Health and Science University, Beaverton, Oregon, USA; University of Alabama at Birmingham; Albert Einstein College of Medicine

**Keywords:** human cytomegalovirus, embryonic stem cells, huNSG mice, latency, monocytes, reactivation, stem cells

## Abstract

Human cytomegalovirus (HCMV) is a highly prevalent beta-herpesvirus and a significant cause of morbidity and mortality following hematopoietic and solid organ transplant, as well as the leading viral cause of congenital abnormalities. A key feature of the pathogenesis of HCMV is the ability of the virus to establish a latent infection in hematopoietic progenitor and myeloid lineage cells. The study of HCMV latency has been hampered by difficulties in obtaining and culturing primary cells, as well as an inability to quantitatively measure reactivating virus, but recent advances in both *in vitro* and *in vivo* models of HCMV latency and reactivation have led to a greater understanding of the interplay between host and virus. Key differences in established model systems have also led to controversy surrounding the role of viral gene products in latency establishment, maintenance, and reactivation. This review will discuss the details and challenges of various models including hematopoietic progenitor cells, monocytes, cell lines, and humanized mice. We highlight the utility and functional differences between these models and the necessary experimental design required to define latency and reactivation, which will help to generate a more complete picture of HCMV infection of myeloid-lineage cells.

## INTRODUCTION

Human cytomegalovirus (HCMV) is a prototypical beta-herpesvirus that infects a majority of the world’s population. Although most HCMV infections are asymptomatic in healthy individuals, the virus is the leading cause of congenital abnormalities following fetal infection and is a significant cause of morbidity and mortality following hematopoietic stem cell transplant and solid organ transplant (1–6). HCMV infection, like with all herpesviruses, is characterized by an initial acute infection followed by the establishment of latency, in which the viral genome is maintained in infected cells without production of new infectious virions. Latency is intermittently broken by periods of viral reactivation, during which viral genes are reexpressed and infectious virus is produced and shed for spread to new hosts. While the interplay between virus and host are key to the control of latency, how these interactions regulate latency establishment and viral reactivation is still unclear.

**FIG 1:**
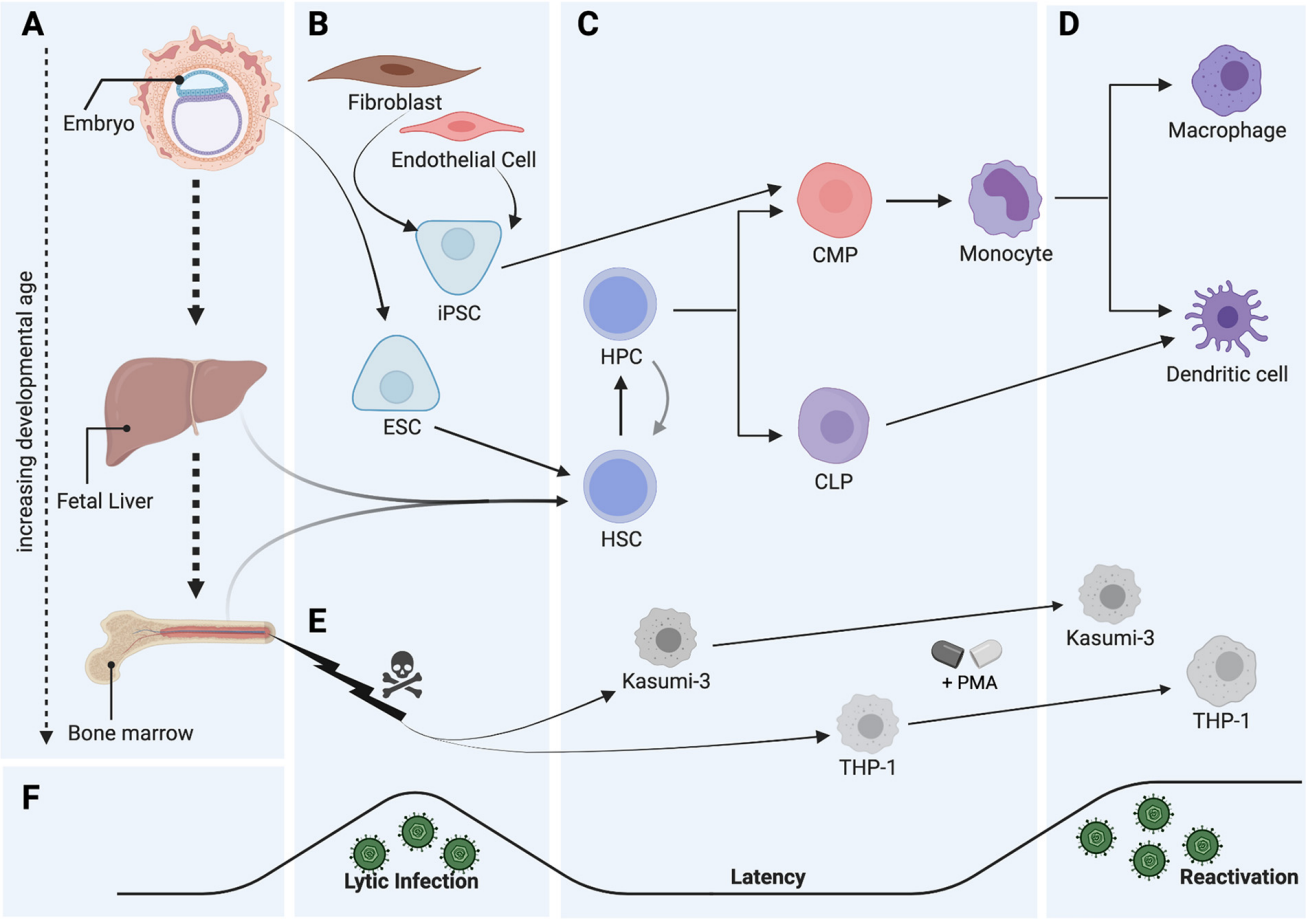
Hematopoiesis and human cytomegalovirus (HCMV). (A) During development, hematopoiesis transitions from the early embryonic yolk sac into the fetal liver and then progresses to the bone marrow just before birth, populating the periphery (including cord blood) along the way. (A, B) Hematopoietic stem cells (HSCs) and hematopoietic progenitor cells (HPCs) can be isolated from tissues (fetal liver, cord blood, or bone marrow) (A) or differentiated from embryo-derived embryonic stem cells (ESCs) (B) or differentiated from induced pluripotent stem cells (iPSCs) derived from mature fibroblasts or endothelial cells (B). (C) Pluripotent HSCs are part of a larger pool of progenitors that can be infected with HCMV. HCMV specifically manipulates progenitors and myeloid lineage cells to control cell fate and create a proviral environment. Differentiation, both normal and driven by HCMV, occurs through a series of progenitors (including the heterogeneous HPC population), which subsequently give rise to either the common myeloid progenitor (CMP) or common lymphoid progenitor (CLP), which can then further differentiate into mature immune cells (monocytes, dendritic cells, and the lymphoid lineage [not shown]). (D) Myeloid differentiation then transitions from the CMP through monocyte stages in the bone marrow followed by trafficking to the periphery and maturation into tissue macrophages. (E) Perturbation of these systems, such as oncogenesis, can lead to cellular transformation, giving rise to transformed cell lines of bone marrow origin (Kasumi-3 and THP-1). These cell lines have been useful as models to study HCMV, including aspects of viral latency as well as subsequent reactivation following treatment with phorbol 12-myristate 13-acetate (PMA). (F) The HCMV life cycle is characterized by an initial acute infection (F, B) followed by latency establishment (F, C), where the virus is maintained for the lifetime of the host. Latency is intermittently broken by periods of viral reactivation (F, D), wherein viral genes are reexpressed and infectious virus is produced for spread to new hosts. Importantly, specific cell lineages naturally and experimentally support different stages of the viral life cycle. This figure was created using BioRender.

In patients, CD34^+^ hematopoietic progenitor cells (HPCs) are a critical reservoir of latent HCMV (7–9) and infection of HPCs contributes to the hematopoietic abnormalities observed following transplantation (10–13). Reactivation *in vivo* is initiated when infected HPCs exit the bone marrow in response to cytokine/growth factor signaling, traffic to the periphery, and differentiate first into monocytes and ultimately into tissue macrophages that support lytic replication (Fig. 1) (10, 14, 15). In contrast to other herpesviruses, like Epstein-Barr virus and Kaposi’s sarcoma-associated herpesvirus, for which latent infection is the default and model systems are relatively well developed, studying the mechanisms of HCMV latency has been difficult due to the lack of appropriate model systems and the inherent difficulty in culturing hematopoietic stem and progenitor cells. Recent years have seen the development of both *in vitro* and *in vivo* models that take advantage of the latest innovations in stem cell culture and genetically engineered mouse strains. Use of these models requires careful consideration of the distinct cell types that harbor latent viral genomes, the systems used for cell culture, and the methods of detecting and measuring virus, all of which are key to understanding HCMV latency. Current controversy surrounding the role of viral gene products in latency establishment, maintenance, and reactivation should be considered in light of these important factors. Here, we will discuss the methodological details important for comparing data generated from various model systems used to evaluate HCMV latency including CD34^+^ HPCs, CD14^+^ monocytes and cell lines, and humanized mice.

## *IN VITRO* MODELS OF HCMV LATENCY AND REACTIVATION

### Primary CD34^+^ hematopoietic progenitor cells.

CD34^+^ HPCs are the latent reservoir of HCMV *in vivo* and, as such, represent the most relevant cell type to investigate both the entry into and the exit from latency *in vitro*. Although direct infection of CD34^+^ HPCs was first reported in 1992 (16), the first model for long-term culture and maintenance of progenitor cell phenotype during *in vitro* HCMV latency was developed in 2002 using adult bone marrow CD34^+^ cells directly infected with laboratory strains of HCMV and cultured on irradiated stromal cells (17). In this model, expression of green fluorescent protein (GFP) driven by the SV40 promoter waned within 4 days of infection, but viral DNA was maintained, and full virus reactivation was shown after stimulation of HPCs with granulocyte colony-stimulating factor (G-CSF) and granulocyte-macrophage colony-stimulating factor (GM-CSF) and coculture on permissive fibroblasts. This model recapitulates the hallmarks of herpesvirus latency, including dampening of viral gene expression, maintenance of the viral genome, and production of new virions upon reactivation stimuli. More recently, this model (17–19) has also been applied to CD34^+^ HPCs derived from fetal liver (20–22) and cord blood (23–25), as well as human embryonic stem cell (hESC)-derived CD34^+^ HPCs (26), and results have been recapitulated using humanized mouse models of latency (20, 21, 27), validating the relevance of this *in vitro* model system.

There are several key aspects of this model that are necessary to the successful study of HCMV latency in HPCs. Below, we highlight some of the important considerations when using the CD34^+^ HPC model of latency: (1) how culture conditions mimic the bone marrow environment and support the pluripotency of HPCs, (2) how the heterogeneity of the CD34^+^ HPC population can affect experimental outcomes and why the source and purity of the cells is important, and (3) the controls required to demonstrate establishment of latency and reactivation.

First, the ultimate goal of an *in vitro* culture model of HPCs is to mimic the *in vivo* conditions as closely as possible in order to maintain their progenitor cell phenotype, typically through use of cell types that act as surrogates for the bone marrow microenvironment. The gold standard for maintaining the functionality of stem and progenitor cells is to culture HPCs on top of stromal cell lines that provide cytokine support to mimic the bone marrow niche (17, 23, 28). In HCMV latency models, the stromal cells are derived from mouse bone marrow and are engineered to express human interleukin (IL-3), G-CSF, and stem cell factor (SCF) (17). When irradiated, the stromal cells provide a nondividing and therefore stable cellular support structure to function as a surrogate niche environment. In addition, the use of mouse stromal cells minimizes the risk of confounding experimental results from productive infection. While the stromal cell model is more technically complex, this platform best mimics the bone marrow niche by expression and regulation of the cytokines and growth factors required to support all key aspects of HPC biology, including survival as progenitors (self-renewal and proliferation) and subsequent differentiation. Coculturing HPCs with stromal cells is routinely used by stem cell biologists to maintain HPC functionality (17, 23, 28, 29) and thus is the most relevant, albeit still an imperfect, model system to mimic the environment of HCMV latent infection of HPCs in the bone marrow niche.

Cell-free culture systems for maintenance of HPCs utilizing media alone are attractive for their simplicity. More recent advances in cytokine cocktails and supplemented medias provides the promise for hematopoietic cell-only culture systems (30) and have been used to study aspects of HCMV latency *in vitro* (31–35). These culture systems include a variety of commercial medias and cytokines meant to maintain cells in culture but differ in the type of cell supported (progenitors versus mature lineages), degree of proliferation support, and, most importantly, the ability to support the maintenance of stem cell function (30). Critically, HPCs grown solely in cytokine-enriched media do not support long-term *in vivo* reconstitution (36), the key hallmark of pluripotency. In contrast, HPCs grown on stromal cell support can maintain repopulation abilities for weeks (37). Thus, it is clear that HPCs maintained in the absence of stromal cell support are functionally distinct, and through having lost their pluripotency, their intracellular environment and responsiveness to extracellular cues are likely even more distinct from HPCs in their native environment *in vivo*.

Quantitative assays to compare viruses and cellular conditions require prior knowledge of the cell types infected in the hematopoietic cell populations. Infection of myeloid-lineage cells with HCMV *in vitro* is inefficient; generally, less than 50% of cells infected with a virus expressing a fluorescent marker demonstrate fluorescence in the days immediately following infection (17, 38, 39). Thus, cell sorting based on viability, CD34 expression, and a fluorescent marker is critical in order to begin an experiment with a pure population of infected cells and to take into account differences in infection rates between viruses based on, for example, variations in viral titers. In addition, beginning with a population of cells that are 100% infected makes measuring the frequency of reactivation a quantitative and biologically relevant experimental read-out and allows for direct comparison of cells infected with wild-type (WT) and mutant viruses.

The cell surface receptor CD34 is detected on a wide range of different progenitor cell types (40), and the importance of cell sorting in latency assays is further underscored by the finding that HCMV infection of different subpopulations of CD34^+^ HPCs results in different functional outcomes. The HSC-like population (CD34^+^CD90^+^CD38^−^) supports a higher frequency of persistent infection and does not establish latency (7, 26). In contrast, slightly more mature stem cell populations expressing CD38 (both CD34^+^CD90^+^CD38^+^ and CD34^+^CD90^−^CD38^+^) can establish latency and reactivate, albeit with different reactivation frequencies (26). Thus, the interaction of HCMV with different progenitor cell subsets is functionally distinct, and there is still much to learn about the biology of the virus in different subsets of CD34^+^ HPCs. Isolation of cells from primary donors, whether fetal liver, cord blood, or adult bone marrow, can result in significantly different proportions of hematopoietic subpopulations (41, 42). In addition, the physical source of the original cell influences its fate, abilities, and likely the outcome following HCMV infection. For example, as the donor ages, both the *in vitro* differentiation potential (43) and *in vivo* reconstitution potential decrease (42, 44), and in parallel, the frequency of HSCs with myeloid bias increases (45). Therefore, the origin or source of HPCs has the potential to greatly influence the biology of both the cell and virus.

Defining when and if a virus has established latency is another important consideration in models of HCMV latency. In our hands, some infectious virus is still present at 7 days postinfection in a stromal cell support model of latency in CD34^+^ HPCs. Thus, a key experimental control in latency studies is to assay for infectious virus directly in parallel with the time point chosen for reactivation. Infectious virus produced during the latency culture likely represents either (1) a subpopulation of lytically infected cells, (2) that culture conditions have resulted in changes in the cell population(s) that now support persistent infection, or (3) that the specific virus being tested is unable to establish latency. To test for the presence of infectious virus immediately prior to reactivation (i.e., virus produced during the latency period: the reactivation control), half the cells are lysed and plated onto permissive fibroblasts. The frequency of infectious virus production is then measured by extreme limiting dilution assay (ELDA). ELDA is a mathematical improvement to limiting dilution assays that provides one-sided confidence intervals for populations that produce 0% or 100% responses. ELDA was developed for the study of stem cell populations in which cell numbers follow non-Poisson distributions and utilizes statistical methods that accommodate small numbers of replicates (46) and as such is appropriate to calculate the low frequencies of reactivation in the limited number of latently infected cells that are used in this model system. Direct comparison of the frequency of infectious virus produced from control and cells stimulated to reactivate determines whether the virus established latency and was capable of reactivation. Cell source, donor-to-donor variation in the proportion of CD34^+^ subpopulations, and culture conditions can all result in different frequencies of reactivation in both the control and reactivated cell populations between experiments. In order to control for these variables, it is most appropriate to combine results from multiple experiments using fold change normalized to the reactivated cell population within each biological replicate. This is a more accurate representation of the data in comparison to fold change over the prereactivation control, which masks the rate of background lytic replication in a given experimental setting. These considerations highlight the importance of culture conditions and experimental design in assessing reactivation from latently infected cells.

The experimental design and controls outlined above are critical to producing high quality data for studying mechanisms of HCMV latency and reactivation. Both the source of CD34^+^ HPCs and their culture conditions are important contributors to the outcome of HCMV latency assays. Therefore, it is unsurprising that differences in experimental outcomes have been documented using different latency systems. As one example, conflicting data about the role of the HCMV-encoded G protein-coupled receptor US28 in latency and reactivation has been reported. US28 is expressed in naturally infected peripheral blood cells (47) and in CD34^+^ HPC and monocyte models during latency (17, 48–51). Using the latency and reactivation model described above and a variety of US28 mutants, we previously demonstrated that US28 ligand binding activity is required for latency in both CD34^+^ HPCs and humanized mice (see below) (21). These results are in contrast to other published data suggesting that in CD34^+^ HPCs US28 constitutive signaling is required for latency establishment and virus-mediated reprogramming of infected cells (38, 51). These discrepancies directly highlight the different possible outcomes of HCMV latency and reactivation from different experimental conditions and underscore the importance of clearly defining and describing the source, identity, and purity of CD34^+^ HPCs, the conditions for culture, and the need for appropriate controls, especially when comparing results across different studies.

### Embryonic stem cell-derived HPCs.

ESCs (Fig. 1A) are pluripotent stem cells capable of self-renewal and differentiation into all three germ layers and have been used to study differentiation and commitment of multiple cell lineages (52, 53). Since ESCs can be maintained as pluripotent cells in long-term culture (54), they can function as an essentially unlimited pool of genetically identical cells, directly avoiding the donor-to-donor variation seen with primary HPCs. Moreover, a cohort of established hESC lines are both National Institutes of Health (NIH)- and institutional review board (IRB)-approved.

hESCs can be directly infected with HCMV, as first shown by the Kalejta group in 2013 (55), although HCMV does not establish latency in these cells at the embryonic stage (Fig. 1B). We differentiated two different NIH-approved hESC lines (WA01 and WA09) into CD34^+^ HPCs (Fig. 1B and C) and utilized the latency model established for primary CD34^+^ HPCs (as described above) to assess the ability of hESC-derived CD34^+^ HPCs to support HCMV latency and reactivation (26). ESC-derived CD34^+^ HPCs in this system can be infected with HCMV and support all aspects of HCMV biology that were tested, including (1) establishment of latency, (2) maintenance of the viral genome, (3) reactivation of infectious virus following stimuli, (4) recapitulation of the function of viral gene products using recombinant viral mutants, and (5) induction of viral-induced myelosuppression via upregulation of TGF-β (26).

Since ESCs are derived from blastocyst stage cells, they are capable of long-term culture but care must be taken to avoid spontaneous differentiation and accumulation of chromosomal abnormalities and mutations. Since ESC differentiation is controlled in the laboratory, differentiation of hESCs to specific HPC or monocytic cell types can be directly regulated, which allows for refined studies on HCMV latency and reactivation in specific cell subsets. On the other hand, since the differentiation method influences final cell type, specific care must be taken to clearly define the cell type being studied and the manner in which they are cultured. Additionally, culture and differentiation of ESCs is time- and labor-intensive, requiring daily cell maintenance. Ideally, improvements in ESC differentiation techniques will refine the ability of these cells to model primary cell function, including engraftment *in vivo*, which is currently lacking.

### Primary CD14^+^ monocytes.

The discovery of CD14^+^ monocytes as a site of HCMV latency in the periphery came from the understanding that HCMV can be transmitted by blood products from healthy seropositive individuals but that viral transmission is reduced through leukocyte depletion (56). Subsequently, CD14^+^ monocytes isolated from seropositive donors were found to contain HCMV genomes (57, 58) and are thought to represent cells derived from latently infected progenitors that are differentiating and have exited the bone marrow. As CD14^+^ monocytes can be easily isolated from venous blood, direct infection of these cells has been used to develop a model of latent HCMV infection. CD14^+^ monocytes have only a short half-life in circulation (1 to 3 days); however, direct infection of monocytes with HCMV has been shown to prolong the life of the cells by induction of specific antiapoptotic and proautophagic signaling (59, 60). Intriguingly, some groups have shown that delivery of HCMV DNA to the nucleus of primary CD14^+^ monocytes is delayed, occurring only after 3 days of infection, and the signaling events initiated by viral binding and entry modulate the intracellular environment in a manner that prevents virus degradation (61). Entry into monocytes significantly differs from more immature progenitor cells in which viral genome delivery occurs within hours and is influenced by cellular signaling pathways in a cell type-specific manner (62). These findings suggest that viral genomes are delivered to distinct intracellular environments following initial infection of CD14^+^ monocytes in comparison to CD34^+^ HPCs, and thus direct infection of CD14^+^ monocytes is unlikely to recapitulate the physiological means by which HCMV establishes latency *in vivo*. Studies have shown that the initial signaling events that occur upon virus binding and entry into CD14^+^ monocytes aids in delivery of the viral genome to the nucleus (61, 63), prevents the induction of apoptosis (64), and triggers the formation of repressive chromatin around the major immediate early promoter (MIEP) to aid in latency establishment (65).

Reactivation from latency is intimately linked to cellular differentiation, and the viral and cellular factors that drive myeloid differentiation play critical roles in the reactivation process. In CD14^+^ monocytes, cellular differentiation activates cellular signaling pathways that give rise to changes in the posttranslational modifications on histones surrounding the immediate-early (IE) promoter, resulting in increased IE gene expression and ultimately production of new virus (65–67). Since cells differentiating down the myeloid lineage (Fig. 1C and D) transit through a number cell states, it is possible that viral gene products function differently in these different cellular environments. Thus, it remains critically important to carefully define the question being addressed in the specific model system being used.

Latent infection of CD14^+^ monocytes provides a useful model to address questions surrounding the effects of viral gene products on driving differentiation from monocytes to macrophages or dendritic cells. In addition, since monocytes are direct players in the immune response, latently infected CD14^+^ monocytes provide a relevant platform to study HCMV’s interaction with the immune system. This is especially relevant given that monocytes consistently make up ∼10% of the circulating hematopoietic cells in the periphery in humans (68) and that reactivation of virus from infected monocytes directly induces changes in T-cell responses *in vitro* (69–72). Thus, monocytes are key players in the complex interplay between HCMV and hematopoiesis, especially with regard to how HCMV mediates myelosuppression, immune responses, and graft failure.

### Induced pluripotent stem cell-derived monocytic lineage cells.

A more recently developed model for the study of HCMV latency and reactivation in monocytes uses induced pluripotent stem cells (iPSCs) (Fig. 1B). iPSCs can be established from adult skin or blood cells, allowing for a renewable, genetically identical source of cells (73). These cells are reprogrammed back to a pluripotent state using forced expression of specific transcription factors and then, similar to ESCs, can be differentiated into many different cell types, including hematopoietic cells (reviewed by Ackermann et al. [74]). Additionally, these cells are amenable to lentiviral transduction and CRISPR/Cas9-mediated gene editing (75, 76), which are powerful tools to assess the role of specific cellular factors in HCMV latency and reactivation (77). Poole et al. assessed the ability of several iPSC lines to differentiate into myeloid cell types (Fig. 1B and C), as measured by cell surface marker expression, and noted significant differences in their ability to support latency via assessment of lytic gene transcripts (78). This again highlights the critical importance of carefully defining the cellular differentiation state and culture conditions used and how these factors can significantly affect myeloid cell and HCMV biology. It should also be noted that both dedifferentiation methods and the cell type of origin for iPSCs influence their fate and function. Further refinements, including less manipulation during dedifferentiation and utilizing iPSCs derived from different cell types (i.e., hematopoietic lineage origin iPSCs, which may be more relevant for HCMV latency), show promise for improving iPSC model function. Both these points are of significant importance when using ESCs and iPSCs that require further differentiation and long-term culture as models for HCMV latency and reactivation.

### Cell line models of HCMV latency.

The use of cell lines to model HCMV latency and reactivation is an attractive option, as cell lines have technical advantages over primary cells, including stability in *in vitro* culture, homogenous genetic background, and ease of manipulation. Because of these advantages, model systems using the myeloid cell line THP-1 (79, 80) and the CD34^+^ myeloblastic cell line Kasumi-3 (81, 82) have been developed (Fig. 1E). These immortalized cell lines can be infected with HCMV and are treated with phorbol esters to induce differentiation and reactivation. However, while these cell lines have been widely used to explore mechanisms of HCMV latency, signaling pathways, especially those involved in cellular differentiation, are dysregulated, and these cells do not exhibit many of the key characteristics of primary cells, such as the ability to form myeloid colonies or engraft in mice (83).

Both THP-1 and Kasumi-3 cells are immortalized human cell lines derived from patients with acute myeloid leukemia (AML). THP-1 cells most closely resemble primary monocytes in both morphology and differentiation properties, while Kasumi-3 cells express CD34 and are more representative of a progenitor cell (79). Both cell lines can be infected with HCMV and show a decrease in immediate early gene expression in parallel with cellular proliferation, although there are conflicting reports as to the time needed to silence viral gene expression in these models. Little infectious virus can be detected in the supernatant of infected THP-1 cells (79), but early studies using Kasumi-3 cells demonstrated ongoing lytic replication unless the cells were treated with phosphonoacetic acid (PAA) (82), a nucleoside analog that inhibits viral DNA replication (84). This suggests that infection of at least a subset of Kasumi-3 cells results in permissive infection, which may be a confounding factor in interpreting experiments using these cells. However, later studies do not report the inclusion of viral DNA replication inhibitors, so the extent of lytic replication and the necessity of drug treatment are unclear. Reactivation is induced in both cell lines by the addition of the phorbol ester 12-*O*-tetradecanoylphorbol-13-acetate (TPA), also known as phorbol 12-myristate 13-acetate (PMA), which promotes differentiation to mature monocytic lineages. An increase in HCMV gene expression can be measured following PMA treatment of THP-1 cells (85, 86), although low titers of new viral progeny have been reported (79, 81), suggesting that THP-1 cells still harbor a defect that prevents efficient viral reactivation. In contrast, viral gene expression and new virions are detected after addition of PMA to infected Kasumi-3 cells (81, 82). Moreover, treatment of these cell lines with PMA prior to exposure to HCMV results in a permissive infection, suggesting that productive replication in these models is dependent on differentiation. Given that the timing of PMA treatment greatly influences the extent of viral gene expression, as well as the differentiation status of these cell lines, it is crucial to include appropriate controls to capture any lytic gene expression and replication in the culture when analyzing latency and reactivation using these models.

While Kasumi-3 and THP-1 cells have significant technical advantages over primary cells, such as the ability to modify these cells by small interfering RNAs (siRNAs) or CRISPR/Cas9, both cell lines have limitations as models for HCMV latency (81, 87). Importantly, dysregulated signaling due to the transformed nature of both cell types presents hazards for studying the cell signaling pathways that regulate latency and reactivation. For example, direct comparison of Kasumi-3 cells to CD34^+^ HPCs found that treatment with HDAC inhibitors had differing effects on IE1 transcription (88, 89). Moreover, THP-1 cells only partially reactivate viral gene expression with limited progeny virus produced after differentiation (80); therefore, these cells may more accurately represent a model of changes in viral gene expression following reactivation stimuli rather than infectious virus production. Overall, the malignant background of Kasumi-3 and THP-1 cells presents a significant risk of experimental bias, and so careful interpretation of data generated using these cell lines is required.

## *IN VIVO* MODELS OF HCMV LATENCY AND REACTIVATION

The strict cellular tropism of HCMV has long been a barrier in understanding viral latency and reactivation *in vivo*. While murine CMV and rat CMV have served as surrogates for *in vivo* studies, they differ substantially from HCMV in their genome organization (90). For instance, they lack homologs of the UL133-138 locus and the RL11 gene UL7, which have been shown to play critical roles during different stages of viral latency and reactivation (20, 27, 91–95). These limitations have driven the development of humanized murine models specific for HCMV in which mice are engrafted with human cells or tissues capable of supporting HCMV infection (reviewed by Koenig et al. [96] and Crawford et al. [97]). While mouse models containing only human fetal tissue xenografts can be infected with HCMV (98, 99), they are not adequate to address questions about latency, persistence, and dissemination because they do not support *de novo* generation of human bone marrow-derived myeloid precursor cells nor mature myeloid lineage cells and do not support viral spread beyond the xenograft tissue.

To overcome the lack of human myeloid precursors in these early models, Smith et al. generated the first humanized mouse model for HCMV in which the mouse bone marrow is engrafted with human CD34^+^ HPCs (100). To do this, they used NSG mice, which allow greater human cell engraftment and function compared to previous immunodeficient mouse strains (101). In this model, adult NSG mice were sublethally irradiated and engrafted with human cord blood CD34^+^ HPCs to generate humanized NSG (huNSG) mice. At 8 weeks postengraftment, approximately 5% of peripheral blood mononuclear cells (PBMCs) were human monocytes (huCD45^+^/huCD33^+^/huCD14^+^), which is approximately half the proportion of monocytes found in the PBMC of healthy humans. Following IP injection of HCMV-infected fibroblasts, HCMV DNA was detectable in samples from organ tissues repopulated with human hematopoietic lineage cells. Based on clinical studies (102–105), Smith et al. (100) hypothesized that G-CSF treatment would promote viral spread to the periphery and trigger HCMV reactivation. Indeed, G-CSF mobilization of huNSG mice robustly increased the percentage of peripheral blood monocytes (to ∼24%) and induced HCMV spread to the peripheral blood, spleen, liver, and kidneys in all tested mice, as well as to the lung, submandibular salivary gland, and bladder in a subset of mice. Moreover, early and late HCMV transcripts were detected in the liver tissue of all HCMV-infected huNSG mice after G-CSF mobilization but were absent in HCMV-infected, nonmobilized huNSG mice. Importantly, immunofluorescence staining of liver tissue revealed that HCMV early and late proteins colocalized exclusively with human monocyte and macrophage markers, demonstrating that HCMV infection occurs only in the human cells repopulating the huNSG mouse. This humanized mouse model is so far the only system for elucidating the role of HCMV gene products during latency and reactivation *in vivo* and for determining their influence on hematopoiesis (20, 21, 27, 92, 106). The strength of this model is the ability to evaluate the physiological role of viral genes involved in latency and reactivation and the ability to highlight differences between the role of HCMV genes *in vitro* versus *in vivo*. For instance, combining studies in humanized mice with *in vitro* analysis in endothelial cells and CD34^+^ HPCs demonstrated that different UL136 isoforms exhibit cell type-specific phenotypes. Specifically, disruption of just the 25-kDa UL136 isoform *in vitro* results in a virus more prone to reactivation, while the same virus fails to reactivate in huNSG mice, suggesting a role for this protein in the context of an intact animal that we do not yet fully understand (27).

Since HCMV latency and reactivation and subsequent disease occur in the context of functional immune responses, Crawford et al. generated a humanized mouse model that is reconstituted with a bone marrow transplant equivalent (CD34^+^ HPCs) in addition to matched human fetal liver and thymus tissue (huBLT) (107). The huBLT mouse model represents a significant improvement over the huNSG model since these mice exhibit increased systemic reconstitution of functional human hematopoietic cells, including myeloid lineage cells, NK cells, and CD4^+^ and CD8^+^ T-cells due, in part, to the presence of human thymic epithelium. This study reported that establishment of a latent HCMV infection of huBLT mice results in the generation of HCMV-specific human CD4^+^ and CD8^+^ T-cell responses, as well as HCMV-neutralizing IgM and IgG antibodies. Upon treatment with G-CSF, the virus reactivates and spreads to peripheral tissues as previously observed in the huNSG model. This model provides a platform to study HCMV latency and reactivation in the context of a functional human immune system and is an excellent tool to understand how HCMV modulates immune responses during latency and reactivation.

Additional studies into the role of HCMV in hematopoietic development have leveraged the huNSG model to elucidate aspects of viral-induced hematopoietic changes *in vivo*. First, Hakki et al. adapted the huNSG model to use G-CSF-stimulated peripheral blood stem cells from HCMV-seropositive donors as the human cell engraftment source (108). We have also utilized the huNSG model to assess the virus-induced changes to the monocytic lineage, including the role of UL7 and US28 in promoting monocyte differentiation *in vivo* (20, 21). Combined, these studies demonstrate the utility of humanized mice to model HCMV-mediated transplant scenarios and to uniquely assess the role of viral gene products *in vivo*.

Overall, the HCMV humanized mouse models have been instrumental in validating viral factors important to latency and reactivation *in vivo* and can be used for understanding the role of key host factors and signaling pathways manipulated by the virus for persistence. Furthermore, these models have been useful to study the effects of viral gene products on maturation of different hematopoietic cell populations. Further development of humanized mouse models with more robust human immune system function and engraftment of additional cell types beyond the hematopoietic lineage (including endothelial and epithelial cells) will open new avenues for investigation to better understand the intricate and balanced relationship between HCMV and its host.

## CONCLUSIONS AND FUTURE PERSPECTIVES

The study of HCMV latency and reactivation in myeloid-lineage cells is fraught with potential pitfalls as cellular differentiation state, virus genetic background, and culture conditions can all have significant impacts on experimental outcomes. These challenges make comparison of HCMV latency studies difficult without a precise understanding of the cell type, culture conditions, and experimental controls used. When designing an HCMV latency experiment, it is important to choose the model system that best addresses the state of latency to be measured (Fig. 1F). Infection of CD34^+^ HPCs is necessary to investigate the role of viral and cellular gene products in latency establishment and maintenance (Fig. 1C and F), as well as those involved in cellular differentiation and viral reactivation, (Fig. 1D and F), while infection of CD14^+^ monocytes can be used to investigate gene products that drive differentiation from monocytes to macrophages or dendritic cells (Fig. 1C and F), those required for reinitiation of viral gene expression and production of new virus, or those important for interactions with the host immune system in the periphery (Fig. 1D and F). Studies modeling signaling pathways and viral gene expression in monocytic cell lines must be interpreted with caution but can provide the basis for more precise studies in primary cell types (Fig. 1E). Finally, studying HCMV latency and reactivation in humanized mouse models provides the most detailed understanding of the role of viral gene products in infection, dissemination, and reactivation in an intact host.

The viral and cellular gene products required for all aspects of latency and their mechanisms of action remain an understudied area of HCMV biology. Recent advances in *in vitro* and *in vivo* model systems, coupled with careful experimental design, can provide new and exciting avenues to understand the mechanisms of action of viral gene products and their interplay with host factors important for regulation of HCMV latency and reactivation.
